# Supplementary material for: Experimental Infection of Domestic Pigs (Sus scrofa) with Rift Valley Fever Virus
Source: Viruses. 2023 Feb 16;15(2):545. doi: 10.3390/v15020545 (PMC9964260; doi:10.3390/v15020545)
Supplement: Supplementary file 1 [file viruses-15-00545-s001.zip › viruses-2171876-supplementary.pdf]

Supplementary Materials

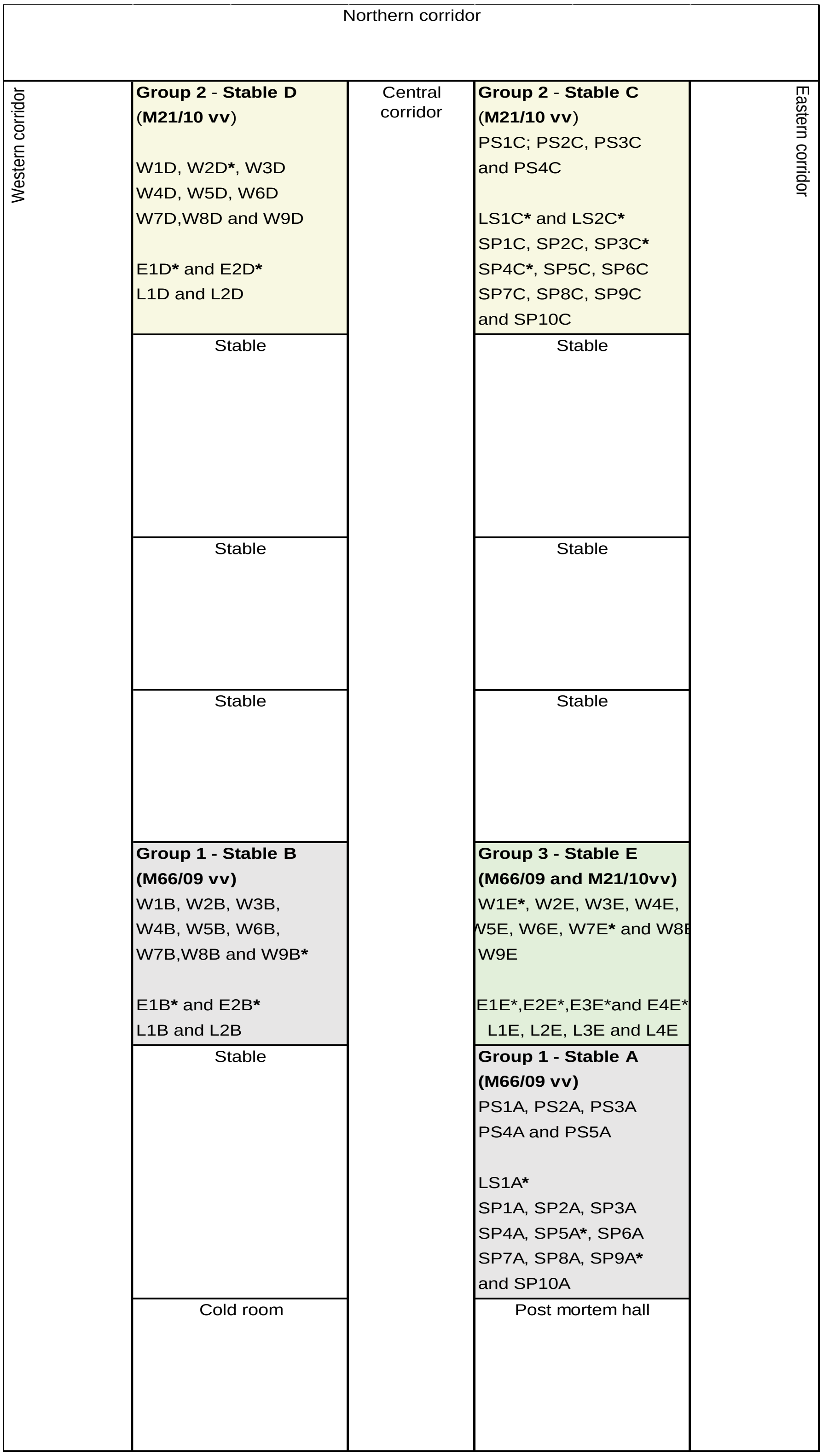

Figure S1: Schematic diagram of the stables in which the experimental animals were housed. Movement was from stables A to B, followed by C to D, then E, using the eastern, western, and northern corridors. The central corridor was used to take dead animals to the post mortem hall or cold room. Proper biosafety and biosecurity procedures were followed and PPE was used for

personnel safety and avoidance of cross contamination. Virus 1 (M66/09 variant) and virus 2 (M21/10 variant) were used to inoculate animals in groups 1 (stables A and B) and 2 (stables C and D) respectively, whereas group 3 animals (stable E) were either inoculated with virus 1 (W2E and W5E) or virus 2 (W4E and W6E) or with a mixture of the two viruses (L1E, L2E, L3E and L4E, and W3E, W8E and W9E). The control piglets and weaners were mock inoculated with TC medium and the ewes and lactating sows received no treatment. The stable codes constituted the animal identity suffixes

Table S1 Laboratory test results of group 1 animals. Only blood and swab pools of animals that demonstrated antibody presence on ELISA were tested on real time RT-PCR (newborn piglets were not swabbed). For blood, swab pools and sera, a negative result represents a collection of negative results of all the samples tested for the particular animal

Animal	Real time RT-PCR			Conventional RT-PCR	ELISA
	Organ pool (DPI)	Blood (DPI)	Oronasorectal swab pool (DPI)	Tissue culture material	Serum (DPI)
PS1A	-	-	+ (3; 4)	+	+ (21; 28; 61)
PS2A	-	-	+ (2; 4)	+	Susp (14); + (21; 28)
PS3A	-	-	-	+	Susp (21); + (28)
PS4A	-	-	-	-	+ (14; 21)
PS5A	-	-	-	+	+ (13; 14)
PS2A-P1	+ (23)	-	N/A	NT	NT
PS2A-P2	-	-	N/A	-	NT
PS2A-P3	+ (23)	-	N/A	-	Susp (23)
PS2A-P4	+ (23)		N/A	-	Susp (23)
PS2A-P5	-	-	N/A	-	Susp (23)
PS2A-P6	+ (23)	-	N/A	-	-
PS2A-P7	-	-	N/A	-	Susp (23)
PS2A-P8	-	-	N/A	+	-
PS2A-P9	-	-	N/A	-	Susp (23)
PS2A-P10	+ (23)	-	N/A	+	-
PS2A-P11	+ (23)	-	N/A	+	-
PS2A-P12	+ (23)	-	N/A	+	-
PS2A-P13	-	-	N/A	-	Susp (23)
PS2A-P14		-	N/A	-	-
PS2A-P15	-	-	N/A	+	NT
PS3A-P1	-	-	N/A	-	Susp (28)
PS3A-P2	N/A	-	N/A	NT	+ (28)
Animal	Real Time RT-PCR			Conventional RT-PCR	ELISA
	Organ pool (DEPI)	Blood (DPI)	Oronasorectal swab pool (DPI)	Tissue culture material	Serum (DPI)
PS3A-P3	-	-	N/A	-	+ (28)
PS3A-P4	-	-	N/A	+	+ (28)
PS3A-P5	+ (28)	-	N/A	+	+ (28)
PS3A-P6	-	-	N/A	-	+ (28)
PS3A-P7	+ (28)	+ (28)	N/A	+	+ (28)
PS3A-P8	-	-	N/A	NT	+ (28)
PS3A-P9	-	-	N/A	-	+ (28)
PS3A-P10	+ (28)	-	N/A	NT	+ (28)
PS3A-P11	+ (28)	-	N/A	+	Susp (28)

PS3A-P12	-	-	N/A	NT	+ (28)
PS3A-P13	-	-	N/A	-	NT
PS3A-P14	-	-	N/A	-	NT
PS4A-P1	+ (32)	-	N/A	-	+ (32)
PS4A-P2	+ (32)	-	N/A	-	+ (32)
PS4A-P3	-	-	N/A	-	+ (32)
PS4A-P4	-	-	N/A	-	+ (32)
PS4A-P5	-	-	N/A	-	+ (32)
PS4A-P6	+ (32)	-	N/A	-	+ (32)
PS4A-P7	-	-	N/A	-	+ (32)
PS5A-AF1 to AF12	+ AF1 (14)<br>+ AF5 (14)	NT	N/A	+ AF5; AF6; AF9	NT
LS1A	NT	NT	+ (5)	NT	+(14)
SP1A	NT	NT	NT	NT	-
SP2A	NT	NT	NT	NT	-
SP3A	- (4)	NT	NT	NT	-
SP4A	+ (4)	NT	NT	NT	-
SP5A*	- (21)	NT	NT	NT	-
SP6A	- (5)	NT	NT	NT	-
SP7A	+ (5)	NT	NT	NT	-
SP8A	- (15)	NT	NT	NT	-
SP9A*	+ (21)	NT	NT	NT	-
SP10A	- (12)	+ (3;4)	+ (4)	NT	+ (14)
E1B	- (29)	NT	NT	NT	-
E2B	- (29)	NT	NT	NT	-
L1B	- (29)	-	+ (1 ; 21)	NT	+ (29)
**Animal**	**Real Time RT-PCR**			**Conventional RT-PCR**	**ELISA**
	**Organ pool (DEPI)**	**Blood (DPI)**	**Oronasorectal swab pool (DPI)**	**Tissue culture material**	**Serum (DPI)**
L2B	+ (29)	-	-	NT	+ (29)
W1B	- (2)	NT	NT	NT	-
W2B	- (4)	NT	NT	NT	-
W3B	- (6)	NT	NT	NT	-
W4B	- (15)	NT	NT	NT	-
W5B	+ (21)	-	+ (3;4)	NT	Susp (14;21)
W6B	- (29)	-	-	NT	+ (21;28;30)
W7B	- (61)	-	+ (21;28)	NT	+ (30)
W8B	- (61)	NT	NT	NT	-
W9B*	- (61)	-	NT	NT	Susp (21)

PS: Pregnant sow; LS: Lactating sow; SP: Suckling piglet; W: Weaner; L: Lamb; E: Ewe; vv: Virus variant; *: Negative control; *: Negative control; NT: Not Tested; N/A: Not applicable; +: Positive; -: Negative; Susp: Suspect; DPI: Days Post Infection; DEPI: Day Euthanised Post Infection or Use.

Table S2 Laboratory test results of group 2 animals. Only blood and oronasorectal swab pools of animals that demonstrated antibody presence on ELISA were tested on real time RT-PCR (newborn piglets were not swabbed). For blood, oronasorectal swab pools and sera, a negative result represents a collection of negative results of all the samples tested for the particular animal

Animal	Real Time RT-PCR			Conventional RT-PCR	ELISA
	Organ pool (DEPI)	Blood (DPI)	Oronasorectal swab pool (DPI)	Tissue culture material	Serum (DPI)
PS1C	-	N/A	N/A	+	-
PS2C	-	-	-	+	Susp (4); + (14; 21; 28; 61)
PS3C	-	-	+ (21)	-	+ (4; 14; 21; 27)
PS4C	-	N/A	N/A	-	-
PS1C-P1	-	-	N/A	-	-
PS1C-P2	+ (27)	-	N/A	+	-
PS1C-P3	+ (27)	+ (27)	N/A	NT	-
PS1C-P4	+ (27)	-	N/A	+	-
PS1C-P5	- (27)	-	N/A	-	-
PS1C-P6	- (27)	-	N/A	-	-
PS1C-P7	+ (27)	-	N/A	NT	-
PS1C-P8	+ (27)	-	N/A	+	-
PS1C-P9	- (27)	-	N/A	+	-
PS1C-P10	+ (27)	-	N/A	-	-
PS1C-P11	+ (27)	-	N/A	-	-
PS1C-P12	+ (27)	NT	N/A	+	-
PS1C-P13	- (27)	NT	N/A	NT	NT
PS1C-P14	+ (27)	NT	N/A	+	NT
PS1C-P15	+ (27)	NT	N/A	NT	NT
PS1C-P16	-	NT	N/A	NT	NT
PS1C-P17	+ (27)	NT	N/A	-	NT
PS2C-P1	-	-	N/A	-	NT
PS2C-P2	-	-	N/A	-	NT
PS2C-P3	-	-	N/A	+	-
PS2C-P4	+ (44)	-	N/A	+	+ (44)
PS2C-P5	-	-	N/A	-	+ (44)
PS2C-P6	+ (44)	-	N/A	+	+ (44)
PS2C-P7	-	-	N/A	+	+ (44)
PS2C-P8	+ (44)	-	N/A	+	+ (44)
Animal	Real Time RT-PCR			Conventional RT-PCR	ELISA
	Organ pool (DEPI)	Blood (DPI)	Oronasorectal swab pool (DPI)	Tissue culture material	Serum (DPI)
PS2C-P9	-	-	N/A	-	+ (44)
PS2C-P10	-	-	N/A	NT	NT
PS2C-P11	+ (44)	-	N/A	NT	NT
PS3C-P1	-	-	N/A	NT	+ (22)
PS3C-P2	+ (22)	-	N/A	-	Susp (22)
PS3C-P3	+ (22)	-	N/A	-	+ (22)
PS3C-P4	-	-	N/A	NT	Susp (22)
PS3C-P5	+ (22)	-	N/A	NT	-
PS3C-P6	+ (22)	-	N/A	-	+ (22)
PS3C-P7	+ (22)	-	N/A	-	+ (22)
PS3C-P8	+ (22)	-	N/A	+	+ (22)

PS3C-P9	-	-	N/A	NT	Susp (22)
PS3C-P10	-	-	N/A	NT	+ (22)
PS3C-P11	+ (22)	-	N/A	-	Susp (22)
PS3C-P12	NT	NT	N/A	+	NT
PS3C-P15	NT	NT	N/A	-	NT
PS4C-P1	-	NT	N/A	NT	-
PS4C-SB2	NT	NT	N/A	-	NT
PS4C-SB3	-	NT	N/A	-	NT
PS4C-P3	NT	NT	N/A		-
PS4C-P4	-	NT	N/A	NT	-
PS4C-P5	+ (32)	NT	N/A	+	-
PS4C-P6	-	NT	N/A	-	-
PS4C-P7	-	NT	N/A	+	-
PS4C-P8	-	NT	N/A	-	-
LS1C	+ (22)	NT	NT	NT	-
LS2C	+ (22)	NT	NT	NT	-
SP1C	NT	NT	NT	NT	-
SP2C	NT	NT	NT	NT	-
SP3C*	- (22)	NT	NT	NT	-
SP4C*	- (22)	NT	NT	NT	-
SP5C	+ (4)	NT	NT	NT	-
SP6C	+ (4)	NT	NT	NT	-
SP7C	NT	NT	NT	NT	-
SP8C	+ (6)	NT	NT	NT	-
**Animal**	**Real Time RT-PCR**			**Conventional RT-PCR**	**ELISA**
	**Organ pool (DEPI)**	**Blood (DPI)**	**Oronasorectal swab pool (DPI)**	**Organ pool (DEPI)**	**Blood (DPI)**
SP9C	NT	NT	+ (6;7)	NT	Susp (1); + (14)
SP10C	- (4)	NT	NT	NT	-
E1D	+ (3)	NT	NT	NT	-
E2D	+ (3)	NT	NT	NT	-
L1D	+ (3)	NT	NT	NT	-
L2D	+ (3)	+ (2)	-	NT	-
W1D	NT	NT	NT	NT	+ (3)
W2D*	- (62)	-	+ (5;7;14)	NT	+ (14;21); Susp (28)
W3D	- (5)	NT	NT	NT	-
W4D	+ (14)	-	+ (5;6;7)	NT	+ (7;14)
W5D	- (22)	-	+ (5;6;14)	NT	+ (14;21)
W6D	- (30)	+ (1)	+ (4;6)	NT	+ (21;28)
W7D	- (62)	-	-	NT	+ (21;28)
W8D	- (3)	NT	NT	NT	-
W9D	- (62)	+ (21)	+ (5;6)	NT	Susp (14); Pos (21;28)

PS: Pregnant sow; LS: Lactating sow; SP: Suckling piglet; W: Weaner; L: Lamb; E: Ewe; vv: Virus variant; *: Negative control; *: Negative control; NT: Not Tested; N/A: Not applicable; +: Positive; -: Negative; Susp: Suspect; DPI: Days Post Infection; DEPI: Day Euthanised Post Infection or Use.

Table S3 Real time RT-PCR and blocking ELISA results of group 3 animals. For blood, oronasorectal swab pools and sera, a negative result represents a collection of negative results of all the samples tested for the particular animal

Animal	Real time RT-PCR			ELISA
	Organ pool (DEPI)	Blood (DPI)	Oronasorectal swab pool (DPI)	Serum (DPI)
L1E♥	- (29)	+ (29)	+ (1;3;5;7;14)	Susp (5); + (21)
L2E♥	- (29)	-	+ (3)	Susp (5); + (21)
L3E♥	- (29)	-	NT	+ (29)
L4E♥	- (29)	-	NT	+ (5;29)
E1E	- (29)	NT	NT	-
E2E	- (29)	NT	NT	-
E3E	- (29)	NT	NT	-
E4E	- (29)	NT	NT	-
W1E*	+ (29)	NT	NT	-
W2E■	- (29)	+ (5)	-	+ (14;30)
W3E♥	+ (29)	NT	NT	-
W4E▲	- (29)	-	+ (7)	+ (14;30)
W5E■	- (29)	+ (1)	-	Susp (30)
W6E▲	- (29)	NT	NT	-
W7E*	- (29)	-	-	Susp (14); + (30)
W8E♥	- (29)	-	-	+ (30)
W9E♥	- (29)	-	-	-

L: lamb; E: Ewe; W: Weaner; *: Negative control; NT: Not Tested; +: Positive; -: Negative; Susp: Suspect; DPI: Days Post Infection; ♥: Inoculated with virus mixture; ■: Infected with virus 1; ▲: Inoculated with virus 2; DEPI: Day Euthanised Post Infection or Use.

Table S4 Comparison of results of RVFV infectivity experiments in weaners conducted in this study and that of Clarke et al., 2021

Analyte	Sample	Proportion positive (%)		*P* - value
		This study	Clarke et al., 2021	
Antibody	Serum/Plasma	53.63 (n = 19)	100 (n = 6)	0.04<br>(95%CI: -2.6% to 67%)
Virus (isolation)	Organs	NT	0 (n = 6)	N/A
	Rectal swabs	NT	0 (n = 6)	N/A
	Blood/Serum	NT	50 (n = 6)	N/A
	Oronasal swabs	NT	33.33 (n = 6)	N/A
RNA	Blood/Serum	20 (n = 10)	0 (n = 6)	0.256<br>(95%CI: -21.6% to 51%)
	Oronasal/oronasorectal swabs	60 (n = 10)	0 (n = 6)	0.02<br>(95%CI: -11.5% to 83%)
	Organs	11.11 (n = 18)	NT	N/A
RNA	TC material - Serum	NT	50 (n = 6)	N/A
	TC material - oronasal swabs	NT	33.33 (n = 6)	N/A

TC: Tissue culture; NT: Not tested; N/A: Not applicable.
